# Use of Non-Cancer Medications in New Zealand Women at the Diagnosis of Primary Invasive Breast Cancer: Prevalence, Associated Factors and Effects on Survival

**DOI:** 10.3390/ijerph17217962

**Published:** 2020-10-29

**Authors:** Phyu Sin Aye, Oliver W. Scott, J. Mark Elwood, Diana Sarfati, Ross Lawrenson, Ian D. Campbell, Marion Kuper-Hommel, Sandar Tin Tin

**Affiliations:** 1 Epidemiology and Biostatistics, School of Population Health, University of Auckland, Auckland 1072, New Zealand; o.scott@auckland.ac.nz (O.W.S.); mark.elwood@auckland.ac.nz (J.M.E.); s.tintin@auckland.ac.nz (S.T.T.); 2 Te Aho o Te Kahu, Cancer Control Agency, Wellington 6011, New Zealand; diana.sarfati@health.govt.nz; 3 Waikato Medical Research Centre, University of Waikato, Hamilton 3240, New Zealand; ross.lawrenson@waikatodhb.health.nz; 4 Waikato District Health Board, Hamilton 3240, New Zealand; ian.campbell@waikatodhb.health.nz (I.D.C.); marion.kuper@waikatodhb.health.nz (M.K.-H.); 5 Surgery, School of Medicine, University of Auckland, Auckland 1072, New Zealand

**Keywords:** breast cancer, medication use, polypharmacy, survival

## Abstract

Background: Assessing the use of multiple medications in cancer patients is crucial as such use may affect cancer outcomes. This study reports the prevalence of non-cancer medication use at breast cancer diagnosis, its associated factors, and its effect on survival. Methods: We identified all women diagnosed with primary invasive breast cancer between 1 January 2007 and 31 December 2016, from four population-based breast cancer registries, in Auckland, Waikato, Wellington, and Christchurch, New Zealand. Through linkage to the pharmaceutical records, we obtained information on non-cancer medications that were dispensed for a minimum of 90 days’ supply between one year before cancer diagnosis and first cancer treatment. We performed ordered logistic regressions to identify associated factors and Cox regressions to investigate its effect on patient survival. Results: Of 14,485 patients, 52% were dispensed at least one drug (mean—1.3 drugs; maximum—13 drugs), with a higher prevalence observed in patients who were older, treated at a public facility, more economically deprived, and screen-detected. The use of 2–3 drugs showed a reduced non-breast cancer mortality (HR = 0.75, 95%CI = 0.60–0.92) in previously hospitalised patients, with other groups showing non-significant associations when adjusted for confounding factors. Drug use was not associated with changes in breast cancer-specific mortality. Conclusions: Non-cancer medication use at breast cancer diagnosis was common in New Zealand, more prevalent in older and disadvantaged women, and showed no effect on breast cancer-specific mortality, but a reduction in other cause mortality with the use of 2–3 drugs.

## 1. Introduction

Breast cancer is the most common cancer in women in New Zealand, with 3286 new cases in 2017 and an age-standardised rate of 94 women per 100,000 population [1]. Patient survival has improved with advances in cancer care so that five-year survival is now 80% or higher [2]. Prolonged survival, together with population ageing and less healthy lifestyles, poses a risk for multi-morbidities [3] and therefore polypharmacy [4].

The use of multiple medications taken concurrently, defined as polypharmacy [5], is often prevalent in cancer patients [6]. In a study of advanced cancer patients across 11 European countries, patients were medicated with as many as 20 drugs, with a mean of 7.8 drugs, and more than a quarter of total patients used 10 or more drugs [7]. Unnecessary medication use is also common in cancer patients. Previous research reported that 22% to 45% of advanced cancer patients were prescribed at least one unnecessary medication [7,8,9], which poses a risk of adverse events, increased costs, and impacting the wellbeing of cancer patients [6].

Thus, assessing the use of non-cancer medications in cancer patients is important in various aspects. Using prospectively collected population-based data, we explored the prevalence of non-cancer medication use in New Zealand women at diagnosis of primary invasive breast cancer, identified associated factors, and assessed their effect on survival.

## 2. Materials and Methods

### 2.1. Data Sources

The data were obtained from four regional breast cancer registries: Auckland, Waikato, Wellington, and Christchurch. The registry data were linked to three routinely collected national data sources using unique National Health Index (NHI) numbers. These were the Pharmaceutical Collection (PHARMS), which contains dispensing information and medication identifiers from pharmacists for subsidised dispensing [10]; the National Minimum Dataset (NMDS), which contains information on all day-patients and inpatients discharged from both public and private hospitals [11]; and the Mortality Collection, which contains information about all deaths recorded in New Zealand [12].

### 2.2. Patient Data

The study sample comprised all women who were newly diagnosed with primary invasive breast cancer (stage I–IV) between 01 January 2007 and 31 December 2016 (*n* = 14,979). Patients who did not have a record of any first cancer treatment within 1 year after cancer diagnosis (*n* = 494) were excluded, which was mainly contributed by advanced stage (anatomic stage IV) patients. Our final dataset for analysis included 14,485 patients.

#### 2.2.1. Non-Cancer Medication Use

Non-cancer medication use was defined as having a dispensing record of one or more drugs (except oncology agents and dermatologicals) supplied for at least 90 days at a time in a period between one year before the date of cancer diagnosis and the date of first cancer treatment (i.e., surgery, chemotherapy, hormonal therapy, or biotherapy). This information was extracted from the PHARMS records.

#### 2.2.2. Mortality Outcomes

Patients were followed from one year after their date of cancer diagnosis until death or last follow-up. To avoid immortal time bias, patient follow-up started from one year after cancer diagnosis, thereby ensuring that any “immortal time” between cancer diagnosis and first cancer treatment was not misclassified [13,14,15]. Information on deaths from breast cancer and other causes during follow-up was extracted from the regional breast cancer registries and the Mortality Collection.

#### 2.2.3. Other Variables of Interest

Information on patient demographics such as age, ethnicity, health domicile code, and facility (private or public); diagnosis and treatment-related information such as tumour histology, anatomic stage, grade, oestrogen receptor (ER), progesterone receptor (PR), and HER2 status, whether the cancer was screen-detected, types of cancer treatment, and treatment date was extracted from the regional breast cancer registers.

The health domicile codes represent patients’ usual residential address and were mapped onto the 2013 New Zealand Deprivation Index (NZDep) with decile ten the most deprived and decile one the least [16]. Staging was based on the anatomic extent of the disease, histologic grade, and biological subtype according to ER, PR, and HER2 status, defined by the American Cancer Society [17].

Information on previous hospitalisations in the period between five years before and five years after breast cancer diagnosis was extracted from the NMDS. Previous hospitalisations indicate the presence of serious comorbidities that require hospital admission.

### 2.3. Data Analysis

Use of non-cancer medications was conceptualised as the number of drugs used by individual patients, grouped into no drug, one drug, two or three drugs, and four or more drugs, and its prevalence was categorised by patients’ demographic, diagnosis, and treatment-related factors. Single and multi-variable ordered logistic regression models were used to identify the demographic factors associated with non-cancer medication use.

To examine the effect of non-cancer medication use on survival, the Kaplan–Meier analyses and log-rank tests were performed to assess differences in mortality from breast cancer-specific causes and mortality from other and unknown causes across the four categories of non-cancer medication use.

Cox regression analyses were then conducted to explore the effect of medication use on survival in terms of breast cancer and other or unknown causes. The multi-variable models were tested for the proportional hazards (PH) assumption by using Schoenfeld residuals. To comply with the PH assumptions, the Cox regression analyses were performed separately based on the presence of previous hospitalisation history (which indicates presence of severe comorbidities) and were further stratified by variables that violated the PH assumption, which were ER, PR, HER2 status, grade, anatomical stage, and histology. Patient demographics, diagnosis, and treatment-related factors were then included in the models.

The data analyses were conducted using Stata version 16. A two-sided *p*-value of <0.05 was regarded as statistically significant in this study.

### 2.4. Ethics Approval

This study was approved by the Central Health and Disability Ethics Committee (Ref: 19/CEN/4). 

## 3. Results

A total of 14,485 women were included in this analysis. Table 1 presents the demographic and clinical characteristics of the study sample.

### 3.1. Prevalence of Non-Cancer Medication Use

**Figure 1:**
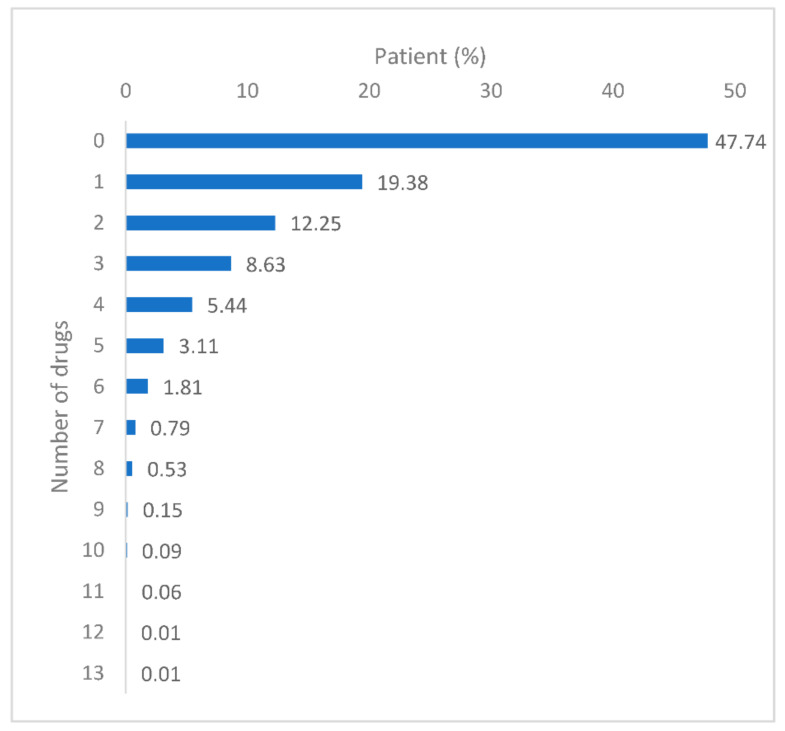
Patient proportions for the number of drugs used.

**Figure 2:**
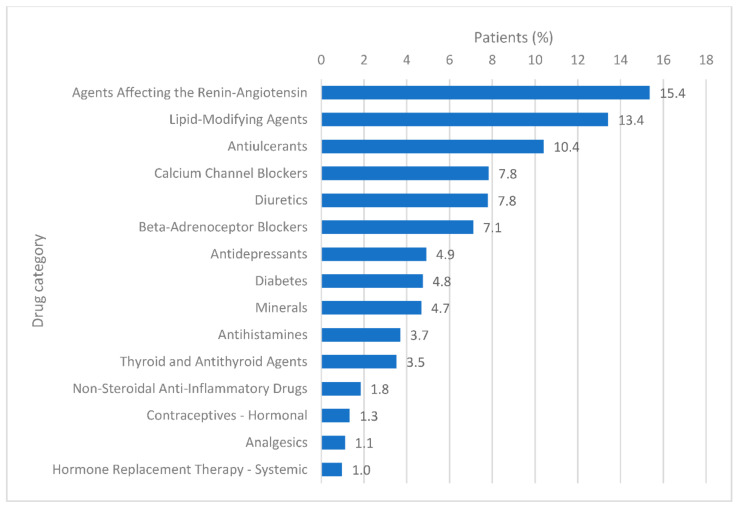
Patient proportions for each drug category, showing the 15 most common categories of a total of 57 categories.

Around 52% of patients used at least one non-cancer medication for a minimum of 90 days between one year before the date of cancer diagnosis and the date of first cancer treatment (Figure 1). The number of medications used ranged from one drug to 13 different drugs (Figure 1), with a mean of 1.3 drugs. The drug categories most commonly dispensed were those of the agents affecting the renin-angiotensin system (*n* = 2911, 15.4%), followed by the lipid modifying agents (*n* = 2543, 13.4%) and anti-ulcerants (*n* = 1971, 10.4%) (Figure 2). The top three drugs dispensed in each of the 15 most common drug categories are shown in Table 2. A detailed report on the number of medications regarding the subgroups of patients is presented in Table 3.

### 3.2. Factors Associated with Amount of Medication Use

The single factor ordered logistic regression showed that the use of a larger number of drugs was significantly associated with older age, European ethnicity, a public facility where first cancer treatment was received, a higher deprivation score, the Christchurch and Waikato regions, and screen-detected cancer in comparison to their respective counterparts (Table 3). The associations remained significant in the multi-variable analysis except for ethnicity.

### 3.3. Effects of Medication Use on Survival

Of the 14,485 women included in this analysis, 813 had been followed for less than one year and were therefore excluded from the survival analysis. During a median follow-up of 4.6 years, 1048 died from breast cancer and 875 from other causes. 

**Figure 3:**
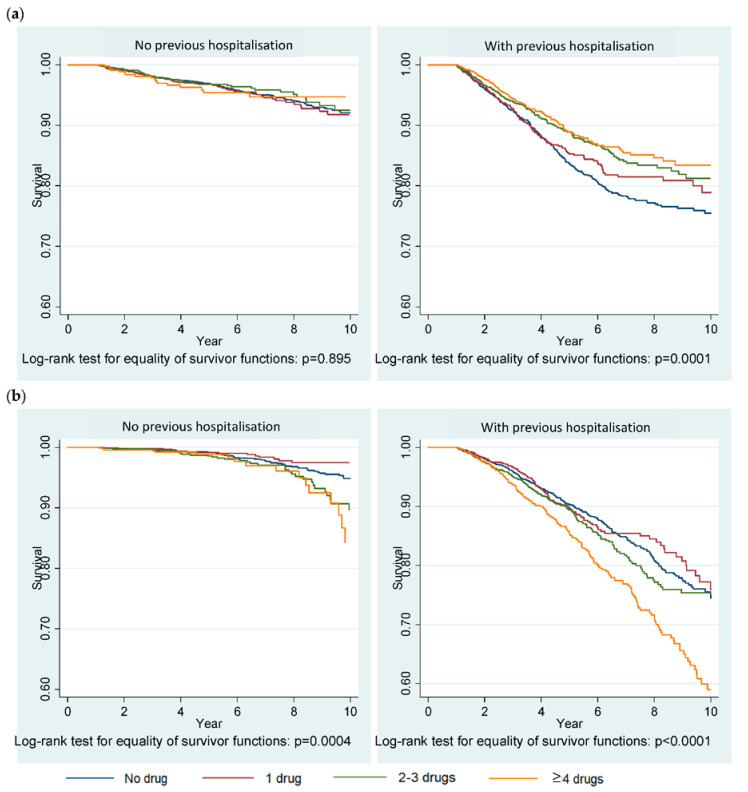
Kaplan–Meier survival graphs showing (**a**) mortality from breast cancer-specific causes and (**b**) mortality from other and unknown causes. Previous hospitalisation indicates presence of a severe comorbidity(-ies) that requires hospitalisation.

The Kaplan–Meier graphs (Figure 3) show significant differences in breast cancer-specific mortality across the different groups of medication use (*p* = 0.0001) in patients with a history of previous hospitalisations, an indicator of the presence of severe comorbidities. Mortality from other and unknown causes differed significantly across the different groups of medication use regardless of the history of previous hospitalisations.

Table 4 reports the results of Cox regression analyses that examined the association between medication use and survival of patients. The single factor analyses (crude effects) support the findings of Kaplan–Meier analysis. In the adjusted models, however, medication use was not associated with mortality from breast cancer in patients with or without previous hospitalisations. Mortality from other and unknown causes, however, significantly decreased with the use of 2–3 drugs (HR = 0.76, 95%CI 0.61–0.94) compared to the “no drugs” group in women with a history of previous hospitalisations.

## 4. Discussion

Our research, to the best of our knowledge, is the first to study the use of medications at the diagnosis of primary invasive breast cancer in New Zealand women. We found that more than half (52%) of the patient population used one or more non-cancer medications, with an average of 1.3 drugs, for at least 90 days within our specified timeframe, exemplifying the frequent use of medications around breast cancer diagnosis. The average number of medications reported in other studies varied widely: 5 to 6.8 depending on age in breast cancer patients [18]; 7.8 in all advanced cancer patients [7]; and as many as 15 in hospice patients, of which one-third had cancer [19]. Due to the varied study population and inclusion criteria of medications, it is complex to compare the results to different studies. Nonetheless, the relatively lower average number of medications in our study was likely because we excluded those dispensed temporarily as well as measuring the use of non-cancer medications up to first breast cancer treatment only.

The number of medications at cancer diagnosis was significantly higher in elderly patients, especially those over 70 years in our study. A previous study of polypharmacy comparing <65 year-old and ≥65 year-old breast cancer patients also observed a significant increase in the prevalence of polypharmacy (50% and 74%, respectively) as well as the average number of drugs (5 and 6.8, respectively) in the elderly group [18]. This pattern also occurs for other types of cancer over the course of the disease and can be explained by a higher number of comorbidities and the complex management of cancer that involves multiple anti-cancer agents and supportive care agents in elderly cancer patients [20]. Our study also showed that patients treated in public facilities, more disadvantaged patients, and those detected through screening used a higher number of medications than their respective counterparts. Medications appeared to be dispensed less to Māori, Pacific, and Asian ethnic groups according to our non-adjusted analysis, but the association became non-significant when adjusted for other factors. A previous New Zealand study indicated that Māori and Pacifica are likely to receive and take less medications for health needs in general [21].

Our study did not show any significant association between medication use and breast cancer-specific mortality but mortality from other and unknown causes was lower in previously hospitalised patients who used 2–3 drugs at cancer diagnosis compared to those who used none. It is likely that the use of 2–3 drugs at cancer diagnosis was more appropriate in patients with severe comorbidities that require hospitalisation. On the other hand, we cannot rule out the fact that the benefits and harms from individual medications may have cancelled each other out. Several studies have reported the benefits from specific medications on cancer survival [22,23,24,25,26] as well as potential adverse effects including various potential drug–drug interactions (PDDI) [27,28] but these are beyond the scope of this paper.

The use of several medications in cancer patients raises several related issues. In a US study of 5490 breast cancer patients aged over 65 years who received intravenous chemotherapy, the median number of prescribed medications for non-cancer reasons prior to cancer treatment was nine, and the number of medications used was related to an increase in post chemotherapy hospitalisation, even after controlling for age, comorbidities, and other factors [29]. In an Australian study, polypharmacy (defined as using five or more medications) was seen in 57% of cancer patients and was associated with being frail rather than robust [30]. Polypharmacy increases the risk of PDDI, which are also related to frailty in breast cancer patients [31], and these include potential interactions with chemotherapy or radiation treatment [32,33]. With multiple medications, there are likely to be potentially inappropriate medications (PIM); studies in the US show that 62% of 17,630 breast cancer patients over age 65 had one or more PIM and had increased inpatient and emergency visits and healthcare costs in the 12 months after cancer diagnosis [34]. PIM were more common than in the age-matched non-cancer subjects [35]. These general issues related to polypharmacy, particularly in elderly cancer patients, show that careful review of medication, particularly prior to specific cancer treatments, and where appropriate, deprescribing to reduce PDDI and PIM, could have considerable benefits [32,33].

We found that simvastatin and omeprazole were most frequently dispensed in our study. Previous studies of polypharmacy in cancer patients also profiled similar drugs of the same drug categories as the most common, besides painkillers [6,18]. While statins are likely to reduce breast cancer recurrence [23] and breast cancer deaths [24] in early diagnosed cases, a large proportion of statin use was deemed unnecessary in patients with limited life expectancy [6]. A multicentre randomised controlled trial in palliative care settings observed that discontinuing statins was not only safe but also associated with improved quality of life and cost saving where approximately half of the patients had cancer [26]. This indicates an opportunity to reduce well-known burdens of polypharmacy in cancer patients, especially in those who are terminally ill.

The key advantage of our study was the use of robust population-based data sources, which include the regional breast cancer registries and the Ministry of Health’s national databases of drug dispensing, hospital discharges, and mortality information. These prospectively maintained breast cancer registry databases contain much more comprehensive and accurate information than national data sources [36,37,38] and capture almost all newly diagnosed breast cancer cases in the respective district health board regions, representing 67% of all breast cancer patients in New Zealand [39]. Our study also had limitations. We aimed to present the burden of non-cancer medications in breast cancer patients, and therefore, we selected the medications that were dispensed for at least 90 days. This excluded the temporary but frequent use of some medications such as painkillers and non-prescription medications, although this limitation also applies to many other studies [29]. We were not able to account for less severe comorbidities that did not require hospitalisation. Our study did not investigate the effect of individual drugs and drug–drug interactions in survival analysis, which may be an opportunity for future research.

## 5. Conclusions

The prevalence of non-cancer medication use is substantial in New Zealand women with newly diagnosed primary invasive breast cancer. Over half of patients used one or more drugs for at least 90 days between one year before cancer diagnosis and first cancer treatment. Medication use was more prevalent in older women and disadvantaged women. The use of 2–3 non-cancer medications at cancer diagnosis reduced mortality from other causes; however, non-cancer medication use had no effect on breast cancer-specific mortality.

## Figures and Tables

**Table 1:** Patient characteristics shown in subgroups.

Subgroups	*N*	%
Total	14,485	100.0
Age		
20–49 yr	4140	28.6
50–59 yr	3903	27.0
60–69 yr	3746	25.9
70–79 yr	1674	11.6
≥80 yr	1022	7.1
Ethnicity		
New Zealand European	10,727	74.1
Māori	1377	9.5
Pacific Peoples	863	6.0
Asian	1190	8.2
Other/Unknown	328	2.3
NZDep2013		
NZDep 1–2 (least deprived)	2582	17.8
NZDep 3–4	2833	19.6
NZDep 5–6	2901	20.0
NZDep 7–8	2177	15.0
NZDep 9–10 (most deprived)	2105	14.5
Unknown	1887	13.0
Region		
Auckland	7962	55.0
Christchurch	2354	16.3
Waikato	2175	15.0
Wellington	1994	13.8
Facility		
Private	5004	34.6
Public	9481	65.5
Screen-detected		
No	8325	57.5
Yes	6160	42.5
Previous hospitalisation (which indicate severe comorbidities)		
No	8138	56.2
Yes	6347	43.8
Time from diagnosis to first cancer treatment		
<31 days	8227	56.8
31–62 days	5215	36.0
>62 days	1043	7.2
Histology		
Ductal	11,170	77.1
Lobular	1683	11.6
Mixed	406	2.8
Other and Unknown	1226	8.5
Anatomic stage		
I	6735	46.5
II	5119	35.3
III	1919	13.3
IV	291	2.0
Unknown	421	2.9
Histologic grade		
1	3267	22.6
2	6534	45.1
3	4300	29.7
Unknown	384	2.7
Biological subtype		
HR+ HER2−	9954	68.7
HR− HER2+	736	5.1
Triple-negative	1498	10.3
Triple-positive	1283	8.9
Unknown	1014	7.0

HR = hormone receptor; HR+ refers to presence of estrogen receptor (ER) and/or progesterone receptor (PR); HR− refers to absence of estrogen receptor (ER) and progesterone receptor (PR).

**Table 2:** Three most common drugs dispensed in each drug category, showing the 15 most common categories of a total of 57 categories.

Three Most Common Drugs in Each Drug Category	*N* of Patients	% within Category
Agents Affecting the Renin-Angiotensin System		
Cilazapril	928	31.9
Quinapril	590	20.3
Cilazapril with hydrochlorothiazide	423	14.5
Lipid-Modifying Agents		
Simvastatin	1508	59.3
Atorvastatin	895	35.2
Bezafibrate	90	3.5
Anti-ulcerants		
Omeprazole	1577	80.0
Pantoprazole	199	10.1
Ranitidine	101	5.1
Calcium Channel Blockers		
Felodipine	774	52.1
Amlodipine	328	22.1
Diltiazem hydrochloride	283	19.1
Diuretics		
Bendroflumethiazide (Bendrofluazide)	940	63.7
Furosemide (Frusemide)	287	19.4
Spironolactone	94	6.4
Beta-Adrenoceptor Blockers		
Metoprolol succinate	920	68.3
Atenolol	187	13.9
Sotalol	62	4.6
Antidepressants		
Citalopram hydrobromide	386	41.5
Fluoxetine hydrochloride	326	35.0
Paroxetine	100	10.7
Diabetes		
Metformin hydrochloride	635	70.5
Gliclazide	145	16.1
Glipizide	93	10.3
Minerals		
Calcium carbonate	428	48.2
Ferrous sulphate	233	26.2
Ferrous fumarate	151	17.0
Antihistamines		
Loratadine	384	54.8
Cetirizine hydrochloride	299	42.7
Promethazine hydrochloride	17	2.4
Thyroid and Antithyroid Agents		
Levothyroxine	631	94.6
Carbimazole	36	5.4
Non-Steroidal Anti-Inflammatory Drugs		
Diclofenac sodium	146	42.0
Ibuprofen	91	26.2
Naproxen	66	19.0
Contraceptives—Hormonal		
Ethinyloestradiol with levonorgestrel	129	51.2
Norethisterone	71	28.2
Ethinyloestradiol with norethisterone	33	13.1
Analgesics		
Paracetamol	144	68.3
Aspirin	37	17.5
Paracetamol with codeine	24	11.4
Hormone Replacement Therapy—Systemic		
Oestradiol valerate	62	33.7
Oestrogens	61	33.2
Medroxyprogesterone acetate	52	28.3

**Table 3:** Prevalence of medication use and associated demographic factors.

		No Drug	1 Drug	2–3 Drugs	≥4 Drugs	Single-Variable Analysis	Multi-Variable Analysis
		*N* (row %)	*N* (row %)	*N* (row %)	*N* (row %)	OR (95%CI)	OR (95%CI)
Total		6915 (47.7)	2807 (19.4)	3025 (20.9)	1738 (12.0)	-	-
Age							
	20–49 yr	2719 (65.7)	873 (21.1)	438 (10.6)	110 (2.7)	1.00	1.00
	50–59 yr	2100 (53.8)	788 (20.2)	726 (18.6)	289 (7.4)	1.75 (1.61–1.91) ***	1.66 (1.52–1.81) ***
	60–69 yr	1393 (37.2)	726 (19.4)	1012 (27.1)	615 (16.4)	3.71 (3.4–4.04) ***	3.44 (3.14–3.76) ***
	70–79 yr	402 (24.0)	272 (16.3)	559 (33.4)	441 (26.3)	7.17 (6.43–7.99) ***	7.21 (6.45–8.05) ***
	≥80 yr	301 (29.5)	148 (14.5)	290 (28.4)	283 (27.7)	6.38 (5.59–7.28) ***	6.47 (5.65–7.41) ***
Ethnicity							
	NZ European	4916 (45.8)	2180 (20.3)	2336 (21.8)	1295 (12.1)	1.00	1.00
	Māori	730 (53.0)	217 (15.8)	258 (18.8)	172 (12.5)	0.82 (0.73–0.91) ***	1.02 (0.90–1.14)
	Pacific Peoples	469 (54.4)	131 (15.2)	162 (18.8)	101 (11.7)	0.77 (0.68–0.88) ***	0.99 (0.86–1.14)
	Asian	650 (54.6)	215 (18.1)	201 (16.9)	124 (10.4)	0.73 (0.65–0.81) ***	1.03 (0.92–1.16)
	Other/Unknown	150 (45.7)	64 (19.5)	68 (20.7)	46 (14.02)	1.04 (0.85–1.27)	1.17 (0.95–1.44)
Facility							
	Public	4408 (46.5)	1737 (18.3)	2007 (21.1)	1329 (14.0)	1.00	1.00
	Private	2507 (50.1)	1070 (21.4)	1018 (20.4)	409 (8.2)	0.79 (0.74–0.84) ***	0.90 (0.84–0.96) **
NZDep2013							
	NZDep 1–2	1291 (50.0)	555 (21.5)	494 (19.2)	242 (9.4)	1.00	1.00
	NZDep 3–4	1348 (47.6)	581 (20.5)	602 (21.3)	302 (10.7)	1.12 (1.02–1.24) *	1.08 (0.98–1.20)
	NZDep 5–6	1321 (45.5)	562 (19.4)	640 (22.1)	378 (13.0)	1.26 (1.14–1.39) ***	1.13 (1.02–1.25) *
	NZDep 7–8	977 (44.9)	409 (18.8)	493 (22.6)	298 (13.7)	1.31 (1.18–1.46) ***	1.21 (1.08–1.35) ***
	NZDep 9–10	1060 (50.4)	341 (16.2)	423 (20.1)	281 (13.4)	1.11 (0.99–1.23)	1.07 (0.95–1.20)
	Unknown	918 (48.7)	359 (19.0)	373 (19.7)	237 (12.6)	1.13 (1.01–1.26) *	0.97 (0.82–1.14)
Region							
	Auckland	3952 (49.6)	1523 (19.1)	1593 (20.0)	894 (11.2)	1.00	1.00
	Christchurch	1003 (42.6)	503 (21.4)	562 (23.9)	286 (12.2)	1.26 (1.16–1.37) ***	1.16 (1.06–1.27) ***
	Waikato	995 (45.8)	383 (17.6)	468 (21.5)	329 (15.1)	1.24 (1.13–1.35) ***	1.10 (1.00–1.21) *
	Wellington	965 (48.4)	398 (20.0)	402 (20.1)	229 (11.5)	1.04 (0.95–1.14)	1.12 (0.97–1.30)
Screen-detected							
	No	4088 (49.1)	1613 (19.4)	1610 (19.3)	1014 (12.2)	1.00	1.00
	Yes	2827 (45.9)	1194 (19.4)	1415 (22.9)	724 (11.8)	1.12 (1.05–1.19) ***	1.21 (1.13–1.30) ***

* *p* ≤ 0.05, ** *p* ≤ 0.01, *** *p* ≤ 0.001; Note: Analysis using ordered logistic regression; the outcome variable is the number of drugs used, grouped into 4 categories: No drug, 1 drug, 2–3 drugs, and ≥4 drugs. Drugs refer to non-anti-cancer drugs dispensed between 1 year before cancer diagnosis date and first cancer treatment date, dispensed for at least 90 days.

**Table 4:** Cox regression survival analysis showing the effects of medication use on mortality from breast cancer-specific causes and other and unknown causes.

Medication Use	No Previous Hospitalisation		with Previous Hospitalisation^	
	Crude HR (95%CI)	Adjusted HR (95%CI)	Crude HR (95%CI)	Adjusted HR (95%CI)
Mortality from breast cancer-specific causes				
1 drug	1.09 (0.81–1.46)	1.07 (0.77–1.47)	0.87 (0.72–1.06)	1.00 (0.81–1.23)
2–3 drugs	0.94 (0.67–1.30)	0.82 (0.56–1.20)	0.72 (0.59–0.87) ***	0.96 (0.78–1.18)
≥4 drugs	1.01 (0.61–1.67)	1.03 (0.58–1.83)	0.66 (0.54–0.82) ***	0.90 (0.71–1.14)
Mortality from other and unknown causes				
1 drug	0.66 (0.39–1.12)	0.69 (0.39–1.20)	0.96 (0.76–1.20)	0.82 (0.65–1.05)
2–3 drugs	1.57 (1.04–2.36) *	1.04 (0.65–1.65)	1.14 (0.94–1.38)	0.76 (0.61–0.94) **
≥4 drugs	2.25 (1.33–3.82) **	0.82 (0.40–1.68)	1.66 (1.38–1.99) ***	0.90 (0.73–1.10)

* *p* ≤ 0.05, ** *p* ≤ 0.01, *** *p* ≤ 0.001; ^ Previous hospitalisation indicates presence of a severe comorbidity(-ies) that requires hospitalisation; the models were stratified by tumour factors such as biological type, histologic grade, anatomic stage and histology and adjusted for age, ethnicity, facility, NZDep2013, region, diagnosis to first cancer treatment duration, and screen-detected.
